# Factors Affecting Nonadherence to WHO's Recommended Antenatal Care Visits among Women in Pastoral Community, Northeastern Ethiopia: A Community-Based Cross-Sectional Study

**DOI:** 10.1155/2022/6120107

**Published:** 2022-08-23

**Authors:** Kusse Urmale Mare, Abel Gebre Wuneh, Mubarek Shemsu Awol, Mohammed Ahmed Ibrahim, Molla Kahsay Hiluf, Setognal Birara Aychiluhm, Osman Ahmed Mohammed, Kebede Gemeda Sabo

**Affiliations:** ^1^Department of Nursing, College of Medicine and Health Sciences, Samara University, Samara, Ethiopia; ^2^Department of Public Health, College of Medicine and Health Sciences, Samara University, Samara, Ethiopia; ^3^Department of Biomedical Sciences, College of Medicine and Health Sciences, Samara University, Samara, Ethiopia

## Abstract

**Introduction:**

More than half of the pregnant women in Ethiopia do not receive the recommended number of antenatal care visits. In the Afar region, where women have limited access to healthcare services due to pastoral livelihood, evidence on noncompliance to the adequate number of antenatal care visits is scarce. Therefore, this study was intended to examine the level of nonadherence to the recommended antenatal care visits and its associated factors in the pastoral community of Northeast Ethiopia.

**Methods:**

A community-based cross-sectional study was conducted from 04 February to 22 March 2020 among randomly selected 703 women who gave birth within 24 months preceding the survey in the Transform HDR districts of the Afar region. Data were collected using a pretested structured interviewer-administered questionnaire. Data were entered into Epi-data version 4.2 and finally exported to Stata version 16 for further analysis. Bivariable and multivariable binary logistic regression analyses were done to identify factors affecting the nonadherence to the recommended antenatal care visits. Odds ratio with the corresponding 95% confidence interval were computed and the statistical significance of the explanatory variables was declared at a *p*-value <0.05.

**Results:**

Nonadherence to the recommended antenatal care visits was 57.0% (95% CI: 53.3%–60.7%). Attending primary (AOR (95% CI): 0.39 (0.22–0.72)) and secondary education and above (AOR (95% CI): 0.25 (0.08–0.77)), being married at the age of 18 years or older (AOR (95% CI): 0.48 (0.36–0.71)), and attending antenatal care at a health center (AOR (95% CI): 0.46 (0.26–0.81)) were associated decreased odds of nonadherence to the recommend visits. Moreover, a higher likelihood of nonadherence was found among women from households not possessing communication media (AOR (95% CI): 1.85 (1.18–2.88)) and those who initiated antenatal care attendance during the second (AOR (95% CI): 5.23 (3.54–7.72)) and third trimesters (AOR (95% CI): 8.81 (1.88–41.20)).

**Conclusions:**

Nearly six in ten women do not receive the recommended antenatal care visits, consistent with the national prevalence. Women's education, age at marriage, type of health facility, possession of mass media, and timing of antenatal care attendance were associated with nonadherence to the recommended antenatal care visits. Thus, improving women's literacy, dissemination of information regarding antenatal care through mass media, and strengthening interventions targeted to end child marriage are important in scaling up the level of adherence. Moreover, supporting community-based health education through health extension programs is crucial in reaching women with limited access to mass media.

## 1. Introduction

Worldwide, about 303,000 maternal deaths occur each year due to pregnancy and childbirth complications, of which 99% and 66% occur in resource-limited settings and Sub-Saharan Africa (SSA), respectively [1]. Ethiopia, which contributes 60% of the global maternal mortality burden with the other nine countries [1, 2], has a maternal mortality ratio of 412 per 100,000 live births [3].

Proper use of antenatal care (ANC) service remains a cost-effective intervention in the reduction of maternal and child mortality and morbidities due to pregnancy and childbirth complications [4, 5]. Complete adherence to the World Health Organization's (WHO) ANC recommendations was also linked with decreased risk of neonatal complications [6]. Although ANC alone contributes to a 20% reduction in maternal mortality [7], adherence to timing and number of visits in developing countries is low [8–10].

Globally, about 80% of women receive at least one ANC, and 64% attend the recommended four visits [2]. In Africa, about three-fourths of pregnant women receive at least one ANC [2], and the prevalence of adequate ANC utilization was 58.5% in SSA countries and 53.4% in the Eastern African region [10]. In Ethiopia, noncompliance to the recommended ANC visits ranges from 36.3% to 63.4% [11–15].

Studies in specific settings found that attendance of less than four ANC visits was 75.6% in Amhara [16], 50.1% in Tigray [17], 23% in Wolaita [18], and 44% in rural Southern Ethiopia [19]. Previous studies showed that factors like maternal education, place of residence, wealth, exposure to mass media, pregnancy intention, and history of abortion had a significant effect on women's compliance to the recommended ANC visits [9, 12, 14, 20–22].

In Ethiopia, various interventions have been implemented to improve maternal health in the last two decades. Expansion and upgrading of health facilities [23, 24] and the launching of the Health Extension Program have resulted in increased health service coverage [23]. Besides, women empowerment through educational opportunities and dissemination of health information through different approaches were taken as the strategies for improving the uptake of maternal health services [23]. The role of behavioral, mobile health, and home-visit interventions on maternal compliance to ANC was also explored in some parts of the country [25, 26].

However, despite these efforts, noncompliance to the recommended number of ANC visits at the national level is relatively high. Moreover, there has only been one study on antenatal care utilization in the Afar region. However, this study only included one district and did not examine the level of maternal compliance to the recommended antenatal care and its predictors [27]. In such pastoral areas, where access to health care services is problematic, there is a dearth of evidence on nonadherence to the recommended ANC visits and its associated factors. Thus, this study aimed to assess nonadherence to the recommended ANC visits and associated factors among women in the pastoral community of Northeast Ethiopia.

## 2. Methods

### 2.1. Study Area, Period, and Design

A community-based cross-sectional study was conducted in Afar Regional State from 04 February to 22 March 2020. Afar region is one of the eleven regional states that constitute the Federal Democratic Republic of Ethiopia. It has five zones, thirty-two districts, and three hundred seventy-nine kebeles (the smallest administrative units in Ethiopia). According to the 2007 national census, the total population of the region is estimated to be 1,805,688 with a rural population share of 91.9% and 90% of people live as pastoralists [50]. There is one referral hospital, six zonal hospitals, 74 health centers, and around 400 health posts in the region.

### 2.2. Population, Sample Size, and Sampling Procedure

`All mothers who gave birth within the last 24 months preceding the survey in the project intervention districts of the region were the source population. The study population was all randomly selected mothers in the randomly selected kebeles of the Transform Health in Developing Regions (THDR) intervention districts. Mothers with children aged 24 months and below and those who resided in the study area for at least six months were included in the study. Mothers who were unable to give the required information due to disability and other conditions were excluded from the study.

A sample size of 746 was estimated by Epi-Info version 7.1.1.14 using a single population proportion formula and by assuming the level of nonadherence to the recommended ANC visits to be 36.3% [11], 5% of margin of error (d), 95% confidence level, 10% nonresponse rate, and a design effect of 2. A multistage sampling technique was used to get the required samples from representative kebeles (clusters). In the first stage, nine districts were randomly selected from 15 THDR intervention districts. In the second stage, three kebeles were selected by lottery method from each of the randomly selected districts (with a total of 27 kebeles). Then, the total number of mothers with children aged 0–24 months in each kebele was identified and a complete listing of eligible mothers was done in the selected kebeles. Based on the number of eligible mothers in the selected kebeles, population proportion to size allocation of the sample was undertaken to determine the number of mothers from each kebele. Finally, a simple random sampling technique was used to select mothers from each kebele.

### 2.3. Data Collection Tool and Procedures

Data were collected using a structured questionnaire through face-to-face interviews adapted from the Ethiopian Demographic and Health Survey 2016 and literature [10, 12, 17, 28–30]. The questionnaire was prepared in English and then translated into the local language, Afar'af. Then, the Afar'af version questionnaire was translated back to the English version by another language expert, and both versions were finally checked for consistency. The data collectors were health professionals (diploma nurses) who have previous data collection experience and were fluent in speaking the local language.

### 2.4. Variables and Definitions

The outcome variable for this study was “nonadherence to the recommended ANC visit,” which was measured by asking the study participants for the number of ANC visits attended during their index pregnancy. Thus, women who attended less than the recommended four visits were considered as having “nonadherence,” coded as “1” and those who attended four or more visits were categorized as having “adhered to the recommendation,” coded as “0.” Independent variables included sociodemographic (women's age, education, and occupation, husband's education and occupation, possession of TV/radio, number of under-five children, decision maker on health care use, family size, and income), obstetrics (age at marriage, age at first pregnancy, gravidity, parity, history of abortion and stillbirth, birth order, gestational age (GA) at 1^st^ ANC visit, and husband/family support for ANC), and health facility-related (time taken to reach the nearest health facility and type of health facility for ANC) characteristics.

### 2.5. Data Quality Control

Data collectors were trained by principal investigators for three days on data collection tools and procedures (sampling and interviewing techniques). Supervision of the data collection activities was undertaken by principal investigators and the collected data were checked for completeness, accuracy, and consistency by supervisors on daily basis. The tool was pretested on 5% (37 mothers) of the sample size in another community (Dubti district) of similar characteristics for some modifications before the commencement of the actual data collection. Data were cleaned and cross-checked before entry and analysis.

### 2.6. Data Management and Statistical Analysis

Data were entered into Epi-data version 4.2 and exported to Stata version 16 for further analysis. Descriptive statistics were used to analyze the descriptive data, and the results were presented in text form, frequency tables, and figures. Bivariable binary logistic regression analysis was done, and variables with *P*-value less than 0.25 were considered as the candidate variables for the final model [31].

Finally, a multivariable binary logistic regression analysis was carried out to identify factors associated with nonadherence to the WHO's recommended ANC visits. Adjusted odds ratio (AOR) with the corresponding 95% confidence interval (CI) was computed, and the statistical significance of the explanatory variables was declared at a *P*-value less than 0.05. Multicollinearity between the independent variables was checked using the variance inflation factor (VIF), and the VIF values for the variables included in the final model were less than 10, indicating there was no collinearity between variables [32]. The model fitness test was checked using the Hosmer and Lemeshow goodness of fit test, and the *P*-value of this test was 0.62 suggesting that the model best fits the data [33].

### 2.7. Ethical Approval and Consent

Ethical approval was obtained from the Research Ethical Review Committee (ERC) of Samara University dated 9^th^ January 2020 and numbered ERC 0003/2020. The letter of the permission was received from Samara University and Afar Regional Health Bureau. Written informed consent was obtained from each study participant before the interview. Confidentiality was maintained by excluding personal identifiers of the study participants from the data, and the collected data were not shared with anybody other than the authors mentioned in this work.

## 3. Results

### 3.1. Sociodemographic Characteristics

From the desired sample size of 746, 703 women participated in the study giving a response rate of 94.2%. Nearly, half (350 (49.8%)) of them were between the age of 25 and 35 years, and 134 (19.2%) had a polygamy type of relationship. Concerning education, 613 (87.2%) women and husbands of 510 (73.3%) women in this study had no formal schooling. Besides, more than ninety percent of women were housewives, and 331 (47.6%) of their husbands were pastoralists. More than half (374 (53.2%)) of the women walk for less than 30 minutes to reach the nearest health facility, and the decision on healthcare use for 261 (37.1%) women was made jointly with their husbands. Moreover, sixty-two (15.5%) women lived in households possessing television and/or radio (Table 1).

### 3.2. Obstetric Characteristics

In this study, the mean ages (±SD) at first marriage and first pregnancy were 16.4 ± 1.9 and 17.9 ± 2.1 years old, respectively. It was found that 330 (46.9%) and 496 (70.6%) women got married and had their first pregnancy before the age of 18 years, of which 418 (59.5%) had been pregnant two to three times.

Regarding adverse pregnancy outcomes, 25 (3.6%) and 45 (6.4%) women had a previous history of abortion and stillbirth, respectively. Nearly half, 351 (49.9%) of respondents attended two to three ANC visits, of which 433 (61.6%) initiated ANC during the first trimester. It was also revealed that 588 (83.8%) women attended ANC at health centers, and 597 (84.9%) had a partner and/or family support for ANC attendance (Table 2).

### 3.3. Nonadherence to WHO's recommended ANC visits

In this study, 50 (7.1%) and 351 (49.9%) women attended one and two to three ANC visits during their index pregnancy, respectively. Overall, the prevalence of nonadherence to the recommended ANC visits was 57.0% (95% CI: 53.3%–60.7%). Variation in the level of noncompliance to the recommended visit was observed across women's educational status and age at first pregnancy. For instance, 365 (91.0%) women with no formal education and 312 (77.8%) women who had their first pregnancy before the age of 18 years did not attend the recommended four or more ANC visits.

### 3.4. Factors affecting Nonadherence to WHO's recommended ANC visits

The result of multivariable logistic regression analysis showed that women's education, possession of communication media, age at first marriage, GA at first ANC visit, and place of ANC attendance were significantly associated with nonadherence to the recommended ANC visits. In this regard, women with primary (AOR (95% CI) = 0.39 (0.22–0.72)) and secondary education and above (AOR (95% CI) = 0.25 (0.08–0.77)), those who married at the age of 18 year or older (AOR(95% CI) = 0.48 (0.36–0.71)), and women who attended ANC at health center (AOR (95% CI) = 0.46 (0.26–0.81)) had lower odds of having nonadherence to the recommend ANC visits. On the other hand, women from the households not possessing radio and/or television (AOR (95% CI) = 1.85 (1.18–2.88)) and those who started ANC attendance during second (AOR (95% CI) = 5.23 (3.54–7.72)) and third trimester (AOR (95% CI) = 8.81 (1.88–41.20)) had an increased likelihood of nonadherence (Table 3).

## 4. Discussions

The current study aimed to assess the level of noncompliance to WHO's recommended antenatal care visits and associated factors among mothers in the Afar Region, Northeastern Ethiopia. Accordingly, the overall level of nonadherence to the recommended ANC visits in this study was 57.1% (95% CI: 53.3%–60.7%), which is consistent with the study conducted in five regions of Ethiopia (56.7%) [13]. However, this finding was slightly higher than the results of the studies done in Tigray, Northern Ethiopia (50.1%) [17], and Egypt (51.3%) [30]. Moreover, this finding was greatly higher than the level of noncompliance to recommended ANC reported in the studies done in Ghana (15.2%) [20], Ethiopia (36.3%) [11], Malaysia (37%) [34], Zambia (40%) [28], Sub-Saharan Africa (41.5%) [10], Myanmar (42%) [35], East African (43.6%) [9], and India (47%) [36]. On the contrary, the level of nonadherence to the recommended ANC visits in this study was lower than that reported in the studies done in Ethiopia (63.4%) [12], Bangladesh (68.7%) [29], Rwanda (86.6%) [37], and Sub-Saharan Africa (92.3%) [8]. This discrepancy might be attributable to the methodological and sample size differences and variations in the study time and setting and background characteristics of the study population.

The result of regression analysis revealed significant differences in the likelihood of nonadherence to the recommended ANC visits among women with different sociodemographic and obstetric characteristics. For instance, women who attended primary education and secondary education and above, respectively, had 41% and 75% decreased odds of being nonadherent to the recommended ANC visits compared to women who did not have formal education. This finding was in agreement with the results of the studies in Ethiopia [12, 14, 18], Ghana [20], East Africa [9], and Sub-Saharan Africa [10, 38] that reported women's education as an enabling factor for attending four or more ANC visits. This finding was also supported by other previous studies [9, 13, 28, 39, 40]. This could be explained by the fact that educated women might have a better awareness of the benefits of adequate ANC attendance [41] and thus more likely to adhere to the recommended visits. Moreover, the negative effect of literacy on healthcare-seeking behavior [42, 43] might also contribute to a lower likelihood of noncompliance to the recommended ANC visits among women with formal education.

Age at first marriage was also identified as a significant factor affecting compliance to the recommended ANC visits. In this regard, the odds of receiving less than four visits were decreased by 52% for women who got married at the age of 18 years or older compared with those married before the age of 18 years. This finding was consistent with the result of the study done in twenty Sub-Saharan African countries, which revealed a 25% decrease in the likelihood of adequate antenatal care attendance among this group [44]. Similar association was also reported in the studies conducted in Nepal [22] and India [45]. This might be attributable to the negative consequence of child marriage on women's empowerment [46] which in turn limits their opportunity in accessing healthcare services.

Place of ANC attendance was found to have a statistically significant effect on the recipient of adequate visits. Women who received ANC at health centers were more than 50% less likely to have nonadherence to the recommended visits compared with women who attended ANC at health posts. This might be due to variations in the quality of antenatal care services provided at different levels of facilities. Thus, women who received a good quality service might be satisfied with the service received and thus likely attend the follow-up visits.

Consistent with the finding of previous studies in Ethiopia [11, 13], women from households not possessing radio and/or television were 1.85 times more likely to receive ANC visits below the recommendation compared with those from households owning these communication media. This finding was also consistent with the result of the studies in India [47], Bangladesh [29], and East Africa [9]. This might be because women with exposure to sources of information, mass media in particular, had better access to health information and, therefore, more likely to use the health service [21, 48].

In this study, the timing of ANC attendance was also found as a significant predictor of noncompliance to the recommended visits. For instance, the odds of nonadherence for women who commenced ANC attendance during the second and third trimesters was increased by more than five and eight folds, respectively, compared with those who had their first visit during the first trimester. This result was consistent with the finding of the studies conducted in Ethiopia [13], seven countdown countries [49], and Ghana [20], which revealed a decreased likelihood of completing the recommended visits with an increase in GA at the first visit. This might be because women who had late ANC booking might have infrequent follow-up visits and thus less likely to receive the recommended four or more visits.

### 4.1. Strengths and Limitations

Since a representative sample of districts was included in this community-based study, its findings can be generalized to the entire Afar region. However, there are certain limitations that should be considered when interpreting the results. First, due to the cross-sectional nature of the study design, evidence of the temporal relationship between explanatory and outcome variables cannot be established. Second, the study mainly relied on self-reported information; therefore, interviewer and social desirability bias may have influenced the participant's responses.

## 5. Conclusions

In this study, nearly six in ten women do not receive the recommended ANC visits. Women's education, possession of communication media, age at marriage, place of antenatal care attendance, and the timing of the first visits were the significant factors affecting nonadherence to the recommended visits. Thus, improving women's literacy, dissemination of information regarding antenatal care through mass media, and strengthening interventions targeted to end child marriage are important in scaling up the level of adherence. Moreover, supporting community-based health education activities through health extension programs is also crucial in reaching women with limited access to mass media.

## Figures and Tables

**Table 1:** Sociodemographic characteristics of women who gave birth in the past two years in the pastoral community, Northeast Ethiopia, 2020 (*n* = 703).

Characteristics	Less than 4 ANC visits	At least 4 ANC visits	Total (%)
Women's age			
15–24 years	167 (41.7)	110 (36.4)	277 (39.4)
25–34 years	187 (46.6)	163 (54.0)	350 (49.8)
35–49 years	47 (11.72)	29 (9.6)	76 (10.8)

Marital relation (n = 696)			
Monogamy	322 (81.5)	240 (79.7)	562 (80.8)
Polygamy	73 (18.5)	61 (20.3)	134 (19.2)

Women's education			
No formal education	365 (91.0)	248 (82.1)	613 (87.2)
Primary	28 (7.0)	43 (14.3)	71 (10.1)
Secondary and above	8 (2.0)	11 (3.6)	19 (2.7)

Husband education (n = 696)			
No formal education	303 (76.7)	207 (68.8)	510 (73.3)
Primary	42 (10.6)	55 (18.3)	97 (13.9)
Secondary and above	50 (12.7)	39 (12.9)	89 (12.8)

Women's occupation			
Housewife	62 (15.5)	66 (21.9)	645 (91.8)
Working^*∗*^	339 (84.5)	236 (78.1)	58 (8.2)

Husband occupation (n = 696)			
Unemployed	32 (8.1)	97 (32,2)	129 (18.5)
Pastoralist	229 (58.0)	102 (33.9)	331 (47.6)
Agro pastoralist	33 (8.4)	21 (7.0)	54 (7.8)
Gov't/private employee	67 (16.9)	71 (23.6)	138 (19.8)
Others^*∗*^	34 (8.6)	10 (3.3)	44 (6.3)

Possession of radio/TV			
Yes	62 (15.5)	66 (21.9)	128 (18.2)
No	339 (84.5)	236 (78.1)	575 (81.8)

Number of U5 children			
1	113 (28.2)	91 (30.1)	204 (29.0)
2	217 (54.1)	157 (52.0)	374 (53.2)
≥3	71 (17.7)	54 (17.9)	125 (17.8)

Family size			
2–4	133 (33.2)	100 (33.1)	233 (33.1)
5–7	178 (44.4)	141 (46.7)	319 (45.4)
≥8	90 (22.4)	61 (20.2)	151 (21.5)

Time taken to reach the nearest health facility			
≥30 minutes	191 (47.8)	138 (45.7)	329 (46.8)
<30 minutes	210 (52.4)	164 (54.3)	374 (53.2)

Decision-makers on healthcare use			
Women only	148 (36.9)	106 (35.1)	254 (36.1)
Husbands only	44 (11.0)	33 (10.9)	77 (11.0)
Joint	143 (35.7)	118 (39.1)	261 (37.1)
Other family members	66 (16.4)	45 (14.9)	111 (15.8)

Family income (birr)			
<500	80 (19.9)	35 (11.6)	115 (16.4)
500–1000	110 (27.4)	104 (34.4)	214 (30.4)
1001–2000	130 (32.4)	109 (36.1)	239 (34.0)
>2000	81 (20.2)	54 (17.9)	135 (19.2)

Working^*∗*^ = herding, gov't employee, and merchant; others^*∗*^ = **g**ov't employee, merchant, and farmer; U5 children = **u**nder-five children.

**Table 2:** Obstetric characteristics of women who gave birth in the past two years in the pastoral community, Northeast Ethiopia, 2020 (*n* = 703).

Characteristics	Less than 4 ANC visits	At least 4 ANC visits	Total (%)
Age at first marriage			
<18 year	215 (53.6)	115 (38.1)	330 (46.9)
≥18 year	186 (46.4)	187 (61.9)	373 (53.1)

Age at first pregnancy			
<18 year	312 (77.8)	184 (60.9)	496 (70.6)
≥18 year	89 (22.2)	118 (39.1)	207 (29.4)

Gravidity			
1	47 (11.7)	42 (13.9)	89 (12.7)
2-3	238 (59.4)	180 (59.6)	418 (59.5)
≥4	116 (28.9)	80 (26.5)	196 (22.9)

Parity			
1	50 (12.5)	45 (14.9)	95 (13.5)
2-3	243 (60.6)	197 (65.2)	440 (62.6)
≥4	108 (26.9	60 (19.9)	168 (23.9)

History of abortion			
No	389 (97.0)	289 (95.7)	678 (96.4)
Yes	12 (3.0)	13 (4.3)	25 (3.6)

History of stillbirth			
No	378 (94.3)	280 (92.7)	658 (93.6)
Yes	23 (5.7)	22 (7.3)	45 (6.4)

Birth order			
1	56 (14.0)	48 (15.9)	104 (14.8)
2	72 (17.9)	51 (16.9)	123 (17.5)
≥3	273 (68.1)	203 (67.2)	476 (67.7)

GA 1st ANC			
1^st^ trimester	186 (46.4)	247 (81.8)	433 (61.6)
2^nd^ trimester	201 (50.1)	53 (17.6)	254 (36.1)
3^rd^ trimester	14 (3.5)	2 (0.7)	16 (2.28)

Place of ANC attendance			
Health post	47 (11.7)	25 (8.3)	72 (10.2)
Health center	324 (80.8)	264 (87.4)	588 (83.6)
Hospital	30 (7.5)	13 (4.3)	43 (6.1)

Husband/family support for ANC attendance			
Yes	332 (82.8)	265 (87.7)	597 (84.9)
No	69 (17.2)	37 (12.3)	106 (15.1)

**Table 3:** Factors associated with nonadherence to the recommended antenatal care visits among women in the pastoral community, Northeast Ethiopia, 2020 (*n* = 703).

Characteristics	Less than 4 ANC visits	At least 4 ANC visits	Corollary (95% CI	AOR (95% CI)
Women's age				
15–24 years	167 (41.7)	110 (36.4)	1.00	1.00
25–34 years	187 (46.6)	163 (54.0)	0.76 (0.55, 1.04)	0.68 (0.46, 1.02)
35–49 years	47 (11.72)	29 (9.6)	1.07 (0.63, 1.80)	0.97 (0.51, 1.86)

Women's education				
No formal education	365 (91.0)	248 (82.1)	1.00	1.00
Primary	28 (7.0)	43 (14.3)	0.44 (0.27, 0.73)^*∗*^	0.39 (0.22 0.72)^*∗*^
Secondary and above	8 (2.0)	11 (3.6)	0.49 (0.19, 1.24)	0.25 (0.08 0.77)^*∗*^

Husband education (*n* = 696)				
No formal education	303 (76.7)	207 (68.8)	1.00	1.00
Primary	42 (10.6)	55 (18.3)	0.52 (0.34, 0.81)^*∗*^	0.84 (0.51, 1.38)
Secondary and above	50 (12.7)	39 (12.9)	0.88 (0.56, 1.38)	1.51 (0.88, 2.60)

Possession of radio/TV				
Yes	62 (15.5)	66 (21.9)	1.00	1.00
No	339 (84.5)	236 (78.1)	1.53 (1.04, 2.25)^*∗*^	1.85 (1.18, 2.88)^*∗*^

Walking distance to the nearest health facility				
≥30 minutes	191 (47.8)	138 (45.7)	1.00	1.00
<30 minutes	210 (52.4)	164 (54.3)	0.93 (0.69, 1.25)	0.94 (0.66, 1.33)

Age at first marriage				
<18 years	215 (53.6)	115 (38.1)	1.00	1.00
≥18 years	186 (46.4)	187 (61.9)	0.53 (0.39, 0.72)^*∗*^	0.48 (0.36, 0.71)^*∗*^

Age at first pregnancy				
<18 years	312 (77.8)	184 (60.9)	1.00	1.00
≥18 years	89 (22.2)	118 (39.1)	0.44 (0.32, 0.62)^*∗*^	0.77(0.48, 1.21)

Parity				
1	50 (12.5)	45 (14.9)	1.00	1.00
2-3	243 (60.6)	197 (65.2)	1.11 (0.71, 1.73)	0.98 (0.56, 1.71)
≥4	108 (26.9	60 (19.9)	1.62 (0.97, 2.70)	1.29 (0.65, 2.55)

GA 1^st^ ANC				
1^st^ trimester	186 (46.4)	247 (81.8)	1.00	1.00
2^nd^ trimester	201 (50.1)	53 (17.6)	5.04 (3.52, 7.20)^*∗*^	5.23 (3.54, 7.72)^*∗*^
3^rd^ trimester	14 (3.5)	2 (0.7)	9.29 (2.09, 41.40)^*∗*^	8.81 (1.88, 41.20)^*∗*^

Place of ANC attendance				
Health post	47 (11.7)	25 (8.3)	1.00	1.00
Health center	324 (80.8)	264 (87.4)	0.65 (0.39, 1.09)	0.46 (0.26, 0.81)^*∗*^
Hospital	30 (7.5)	13 (4.3)	1.23 (0.55, 2.76)	0.94 (0.38, 2.30)

Family support for ANC attendance				
Yes	332 (82.8)	265 (87.7)	1.00	1.00
No	69 (17.2)	37 (12.3)	1.49 (0.97, 2.29)	1.03 (0.62, 1.69)

^*∗*^Significant at *P* < 0.05; ANC = antenatal care; COR = crude odds ratio; AOR = adjusted odds ratio; CI = confidence interval.

## Data Availability

The raw datasets' data used to support the findings of this study are freely available from the corresponding author and can be shared upon reasonable request.

## Abbreviations

ANC: Antenatal care
AOR: Adjusted odds ratio
CI: Confidence interval
COR: Crude odds ratio
ERC: Ethical review committee
GA: Gestational age
THDR: Transform health in developing regions
WHO: World health organization.
